# The remnant of the European rabbit (*Oryctolagus cuniculus*) IgD gene

**DOI:** 10.1371/journal.pone.0182029

**Published:** 2017-08-23

**Authors:** Dennis K. Lanning, Pedro J. Esteves, Katherine L. Knight

**Affiliations:** 1 Department of Microbiology and Immunology, Center for Translational Research and Education, Stritch School of Medicine, Loyola University Chicago, Maywood, Illinois, United States of America; 2 InBIO-Research Network in Biodiversity and Evolutionary Biology, CIBIO, Campus de Vairão, Universidade do Porto, Campus Agrário de Vairão, Vairão, Portugal; 3 Departamento de Biologia, Faculdade de Ciências, Universidade do Porto, Porto, Portugal; 4 CITS - Centro de Investigação em Tecnologias de Saúde, CESPU, Gandra, Portugal; Michigan State University, UNITED STATES

## Abstract

Although IgD first appeared, along with IgM, in the cartilaginous fishes and has been retained throughout subsequent vertebrate evolution, it has been lost in a diverse group of vertebrate species. We previously showed that, unlike vertebrates that express IgD, the rabbit lacks an IgD (*Cδ*) gene within 13.5 kb downstream of the IgM gene. We report here that, by conducting BLAST searches of rabbit Ig heavy chain genomic DNA with known mammalian IgD exons, we identified the remnant of the rabbit *Cδ* gene approximately 21 kb downstream of the IgM gene. The remnant *Cδ* locus lacks the *δCH1* and hinge exons, but contains truncated *δCH2* and *δCH*3 exons, as well as largely intact, but non-functional, secretory and transmembrane exons. In addition, we report that the *Cδ* gene probably became non-functional in leporids at least prior to the divergence of rabbits and hares ~12 million years ago.

## Introduction

Since its discovery in human serum in 1965 [1, 2], IgD has remained an enigmatic immunoglobulin (Ig) isotype. It first appeared, along with IgM, at the dawn of adaptive immunity in cartilaginous fishes [3] and has been preserved throughout subsequent vertebrate evolution. While its ancient origin and evolutionary persistence suggest an important immunological role, IgD has clearly been lost in a diverse group of vertebrate species. No evidence or remnant of an IgD (*Cδ*) gene has been found in the Ig heavy chain locus of the chicken [4], duck [5], opossum [6] or rabbit [7, 8]. The guinea pig also lacks a *Cδ* gene, although one remnant fragment of the *δCH2* exon and two remnant fragments of the *δCH3* exon were found lying in reverse transcriptional orientation downstream of the IgM (*Cμ*) gene [9]. Similarly, the Ig heavy chain locus of the African elephant was recently shown to lack a *Cδ* gene, although a short remnant of the *δCH3* exon was identified [10].

Efforts to identify IgD in rabbits began with the discovery that, in addition to humans, IgD is also expressed in mice [11, 12], several nonhuman primates [13] and rats [14], suggesting that IgD might be widely expressed among mammals. Employing approaches similar to those used to identify IgD on mouse lymphocytes, several investigators found evidence for an IgD-like molecule on rabbit lymphocytes. SDS-PAGE analyses of rabbit lymphocyte lysates precipitated with anti-μ antibody (Ab) to remove IgM, and anti-light chain Ab to detect residual Ig molecules, for example, identified a non-γ, non-α Ig molecule that, like human and mouse IgD, was of comparable molecular weight to IgM and proteolytically labile [15, 16]. Similarly, a residual surface Ig molecule, not detectable with anti-μ, -γ or -α antibodies and therefore thought to be IgD, was observed on rabbit mesenteric lymph node lymphocytes that had been depleted of surface IgM by incubation under capping conditions with anti-μ Ab [17].

Despite these promising early results, conclusive evidence for IgD in rabbit remained elusive. A similar inability to definitively identify IgD in several other vertebrate species, despite the advent of powerful molecular approaches, suggested that IgD might be a recently evolved Ig isotype in primates and rodents. The identification of an IgD homolog in teleosts, however, including channel catfish [18], Atlantic salmon [19] and Atlantic cod [20], demonstrated that, rather than a recent evolutionary innovation, IgD is actually evolutionarily ancient. Indeed, the more recent finding that IgD in the amphibian *Xenopus tropicalis* is orthologous to IgW, an Ig isotype found only in cartilaginous fish and lungfish, established that IgD, like IgM, was present in the earliest jawed vertebrates [3]. A modified view, that IgD, although evolutionarily ancient, has been lost in all mammals except primates and rodents [21, 22], was also subsequently dispelled with the identification and characterization of *Cδ* genes in the cow, sheep and pig in 2002 [23], and in a variety of additional mammals since [24–28]. It is now known that the *Cδ* gene is unevenly distributed among mammalian species, present in some and deleted in others, a puzzling situation that challenges efforts to understand its role in adaptive immunity.

In vertebrates that express IgD, the *Cδ* gene is located immediately downstream of the *Cμ* gene, with which it is co-transcribed [29]. We previously determined the nucleotide sequence of 13.5 kb of genomic DNA downstream of the rabbit *Cμ* transmembrane exons and demonstrated that this region contained no evidence of a rabbit *Cδ* gene [7]. Shortly thereafter, Ros *et al*. (2004) determined the nucleotide sequence of 4 BAC clones collectively comprising 0.5 Mb of the European rabbit Ig heavy chain locus and similarly found no evidence of a rabbit *Cδ* gene [8]. Here, we report finding the remnant of the rabbit *Cδ* gene (the CH2, CH3, secretory and transmembrane remnant exons) ~21 kb downstream of the *Cμ* transmembrane exons. Additionally, we report that the *Cδ* gene probably became non-functional in leporids at least prior to the divergence of rabbits and hares ~12 million years ago.

## Materials and methods

### Identification of rabbit Cδ remnant exons and repetitive DNA elements

Genomic nucleotide sequence >13.5 kb downstream of the Cμ transmembrane exons in the European rabbit Ig heavy chain locus, obtained from BAC clone 27N5 (GenBank accession no. AY386696) reported by Ros *et al*. (2004) [8], was used in BLAST searches to identify regions homologous to known mammalian IgD exons. Genomic sequences thus identified were confirmed by alignment with known mammalian IgD exons using CLUSTAL W [30] in the BioEdit software [31] and visual inspection. Repetitive DNA elements (processed pseudogenes, LINEs, C repeats, etc.) were identified by BLAST searches and with the Censor software tool provided by the Genetic Information Research Institute [32].

### PCR amplification of the remnant δCH2 and δCH3 exons from Iberian hare

Touchdown PCR was used to amplify an 1882 bp genomic region containing the remnant *δCH2 and δCH3* exons from Iberian hare (*Lepus granatensis*) and European rabbit (*Oryctolagus cuniculus*; as positive control) splenic DNA using the following primers: RbDelta exon 2 f: 5’-cagacttctggccattgcacttctg-3’; RbDelta exon 3 r: 5’- gacccaagcaaacccagtgacaaac-3’. 100 pg of genomic DNA template was amplified, in the presence of 10% DMSO, under the following conditions: 94°C, 1 min; 59°C, 1 min; 72°C, 3 min, with the annealing temperature decreased 1°C every 2 cycles for 20 cycles, and 15 additional cycles at the final annealing temperature of 49°C; final extension at 72°C, 5 min. PCR product of the appropriate size was gel-purified and ligated into the pGEM-T vector (Promega, Madison, WI). The ligation was transformed into DH10B electrocompetent cells, and plasmids containing the cloned PCR product were identified by blue/white screening on ampicillin-LB agar and colony PCR. Positive colonies were inoculated into 10 ml LB broth cultures with 100 μg/ml ampicillin and shaken overnight at 37°C. Following plasmid isolation, the nucleotide sequences were determined using an automated ABI Prism 310 sequencer with Big Dye-labeled terminators (Perkin-Elmer Applied Biosystems, Foster City, CA). The nucleotide sequence is deposited in GenBank (accession number MF425650).

### Strategies to detect mRNA transcripts containing rabbit Cδ exons

RT-PCR using V_H_1- and Cδ exon-specific primers was used to search for VDJ gene transcripts containing rabbit Cδ2 and Cδ3 exons in rabbit appendix cDNA. The following PCR conditions were used: 94°C, 1 min; 58–60°C, 1 min; 72°C, 3 min; 30 cycles, followed by final extension at 72°C, 5 min. The following primers were used: RbVDJ-Cmu Nest f2: 5’-cctggttcgctgtgctcaaag-3’; RbDelta CH2 r2: 5’-gcccctcccaggacaagtg-3’; RbDelta CH3 r: 5’-ccgctgacctccagcagtttc-3’; a Cμ-specific primer, RbVDJ-Cmu Nest r2, was used as a positive control: 5’-agacgagcgggtacagagttg-3’.

3’ RACE using Cδ exon-specific primers was used to search for VDJ gene transcripts containing rabbit δS and δTM1 exonsin cDNA prepared from rabbit spleen, appendix and bone marrow, as described in [33], using the following primers: RbDelta SecExon 3’RACE f1: 5’-cacagcggctggaagca-3’; RbDelta SecExon 3’RACE f2: 5’-gagctggcaagcagtga-3’; RbDelta TM1 3’RACE f1: 5’-agccccgcagggacaa-3’; RbDelta TM1 3’RACE f2: 5’-gacaacaaaggtgacgactacat-3’; and Q_T_, Q_1_ and Q_2_ RACE primers [33]. Cμ-specific primers, C mu f (5’-gtgagcctgtcatctccaa-3’) and Mid mu f (5’-aagcacaccatctccaagtcca-3’) were used as a positive control.

European rabbit (*O*. *cuniculus*) spleen, appendix and bone marrow samples were obtained in compliance with a protocol approved by the Loyola University Medical Center Institutional Animal Care and Use Committee. Euthanasia was performed by pentobarbitol overdose (150 mg/kg), i.v. Iberian hare (*Lepus granatensis*) and American pika (*Ochotona princeps*) tissue samples were provided by the CIBIO Lagomorpha tissue collection. The Iberian hare was collected in Alcochete, south of Portugal; the identifying number for the spleen sample obtained by the CIBIO Lagomorpha tissue collection was Lgalc5. The American pika was collected in the United States; the identifying number for the spleen sample obtained by the CIBIO Lagomorpha tissue collection was Ocpri1. It was not necessary to obtain approval from an ethics committee for *Iberian hare and Ochotona princeps* because these samples have been described and used in previous publications [34–37].

### Phylogenetic studies

The nucleotide sequences of the rabbit and hare Cδ exon remnants and the Cδ exons of other mammals were aligned using CLUSTAL W [30] as implemented in BioEdit software [31], followed by visual inspection. A Maximum Likelihood approach was used to estimate the phylogenetic relationships between the *Cδ* exon sequences by using MEGA6 [38] with the following options: bootstrap method (1000 replicates), General Time Reversible as model and pairwise deletion for gaps/missing data treatment.

## Results

**Fig 1:**
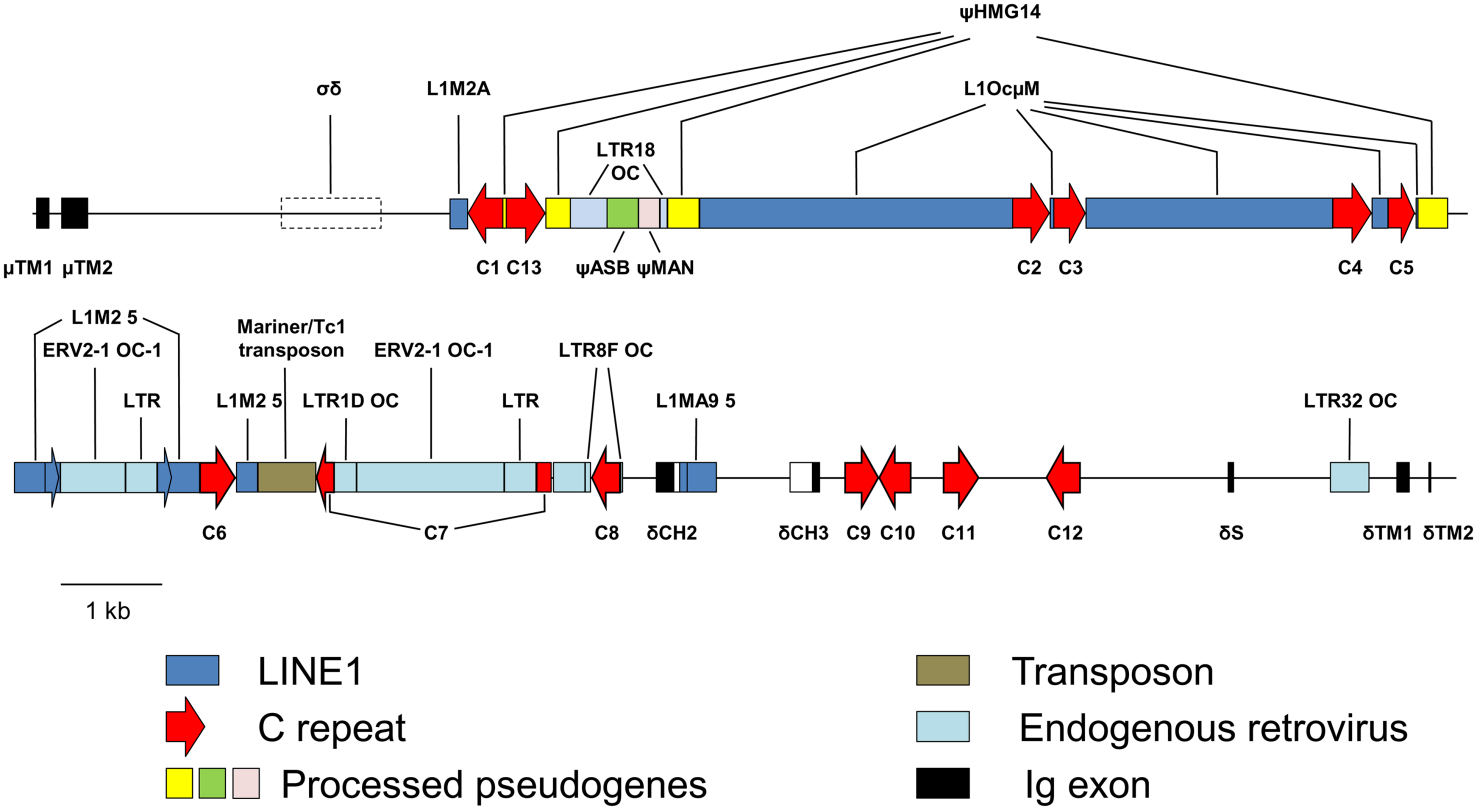
The remnant of the rabbit IgD locus. The remnant of the rabbit *Cδ* gene and the inserted repetitive DNA elements downstream of the *Cμ* transmembrane exons are depicted. The rabbit *Cδ* exons are represented by black rectangles. White rectangles adjacent to the *δCH2* and *δCH3* exons indicate regions of loss of similarity to their homologs in other mammalian species (the non-homologous region of *δCH2* overlaps L1MA9 5). Repetitive DNA elements are labeled and designated by colored rectangles. The directionality of C repeats, and direct repeats flanking the 5’-most endogenous retrovirus, are indicated by arrowheads. The putative σδ switch region is indicated by a hatched rectangle. *δS*, *Cδ* secretory exon; *δTM1*, *Cδ* transmembrane exon 1; *δTM2*, *Cδ* transmembrane exon 2; *μTM1*, *Cμ* transmembrane exon 1; *μTM2*, *Cμ* transmembrane exon 2; ψHMG14, ψASB, ψMAN, high-mobility group 14, arylsulfatase B and endo-alpha-like mannosidase processed pseudogenes, respectively; σδ, putative σδ switch region; nomenclature for C repeats (and the LINE1, L1OcμM) follows that adopted in [7] (C13 was not present in the genomic DNA used in that study); nomenclature of other repetitive DNA elements follows that used by the Genetic Information Research Institute [32].

By conducting BLAST searches with known mammalian IgD exons, we identified the remnant of the rabbit *Cδ* gene within a 9.5 kb region approximately 21 kb downstream of the second *Cμ* transmembrane exon (Fig 1). We found no evidence of the *δCH1* or hinge exons. The genomic region where they would be expected to be found was instead densely occupied by multiple types of repetitive DNA elements (partially described in [7]). These include portions of high-mobility group 14 (ψHMG 14), arylsulfatase B (ψASB) and endo-alpha-like mannosidase (ψMAN) processed pseudogenes, a truncated Mariner transposon, two truncated Long Interspersed DNA Elements (LINEs), two endogenous retroviruses (ERVs), two solo ERV long terminal repeats (LTRs) and eight C repeats (the most common Short Interspersed DNA element (SINE) in the rabbit genome [39]. Four additional C repeats, a truncated LINE and a solo ERV LTR were inserted within the *Cδ* gene remnant. In addition, while the *Cδ* secretory and transmembrane exons were largely intact, though apparently non-functional, only the 3’ two-thirds and 5’ one-quarter of the *δCH2* and *δCH*3 exons, respectively, were identifiable. Furthermore, a potential IgD class switch (σδ) region was identified in the Cμ-Cδ intron 2.3 kb downstream of the μTM2 exon and about 660 bp 5’ of the first repetitive DNA element (Fig 1 and S1 Fig). σδ regions, which mediate rare class switching events in which the Cμ gene is deleted and the Cδ gene is directly joined to the rearranged VDJ gene [40–43], are G-rich and contain relatively high numbers of pentameric repeats, particularly TGGGG, TGGGC and TGAGC. Nineteen such pentamers lie within a 1 kb G-rich region of the rabbit Cμ-Cδ intron, suggesting that the intron might once have contained a functional σδ region able to mediate IgD class switching. The region of the rabbit Ig heavy chain locus that we analyzed is contained in the overlapping BAC clones 219D23 and 27N5 reported by Ros *et al*. (2004). The positions of the remnant *Cδ* exons and neighboring repetitive DNA elements in the nucleotide sequence of BAC clone 27N5 (accession number AY386696) are listed in Table 1.

**Table 1:** Nucleotide positions of rabbit remnant *Cδ* exons and neighboring DNA elements in BAC clone 27N5.

*μTM1*	6933–7051	LINE1	17280–19713	C7	26161–26292
*μTM2*	7178–7443	C4	19714–20100	LTR8F OC	26311–26356
σδ	9365–10353	LINE1	20101–20251	C8	26357–26630
L1MA5	11019–11192	C5	20252–20516	LTR8F OC	26631–26649
C1	11193–11546	LINE1	20517–20538	Cδ2	26984–27162
ψHMG14	11547–11579	HMG14	20539–20839	L1MA9 5	27223–27592
C13	11580–11965	L1M2 5	21039–21493	Cδ3	28552–28624
ψHMG14	11966–12213	ERV 2–1 OC-1	21494–22133	C9	28862–29182
LTR18 OC	12214–12574	ERV 2–1 OC-1 LTR	22134–22438	C10	29183–29495
ψASB	12575–12890	L1M2 5	22439–22851	C11	29802–30141
ψMAN	12891–13098	C6	22852–23190	C12	30796–31116
LTR18 OC	13099–13167	L1M2 5	23191–23396	δS	32567–32615
ψHMG14	13168–13479	HSMAR	23397–23959	LTR32 OC	33558–33937
LINE1	13480–16572	C7	23960–24138	δTM1	34210–34335
C2	16573–16940	LTR1D OC	24139–24363	δTM2	34523–34531
LINE1	16941–16969	ERV2-1 OC-1	24396–25846		
C3	16970–17279	LTR2C OC	25847–26160		

Cμ transmembrane exons, Cδ remnant exons and repetitive DNA elements are listed in consecutive order in columns 1, 3 and 5 with their positions in BAC clone 27N5 listed to their right.

### The rabbit δCH2 and δCH3 exon remnants

**Fig 2:**
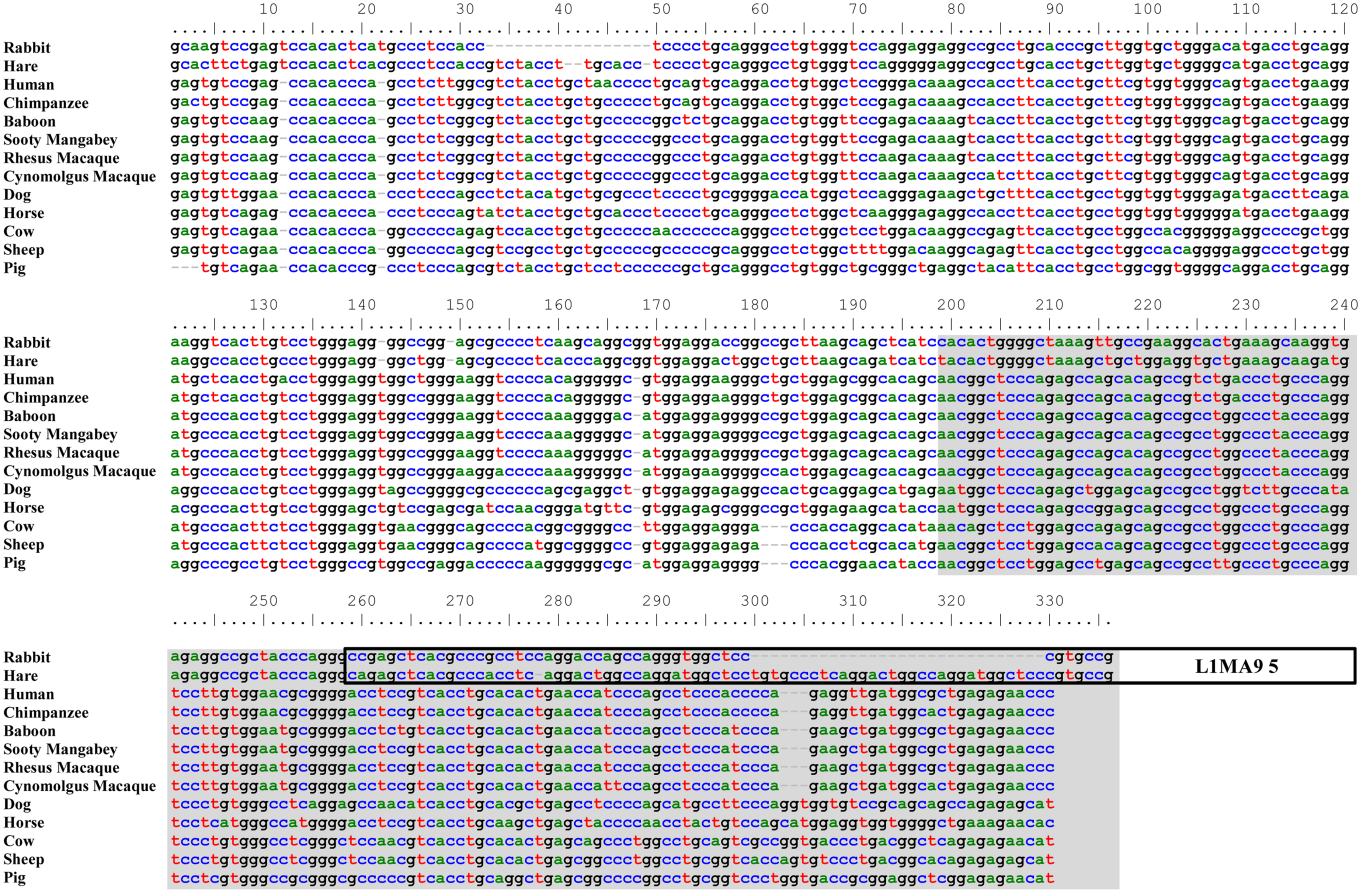
Alignment of rabbit and hare remnant *δCH2* exons with the *δCH2* exons of various mammalian species. Nucleotide positions are numbered above each panel. The region with no similarity to other mammalian *δCH2* exons is highlighted in gray. The region of overlap with L1MA9 5 enclosed in box.

**Fig 3:**
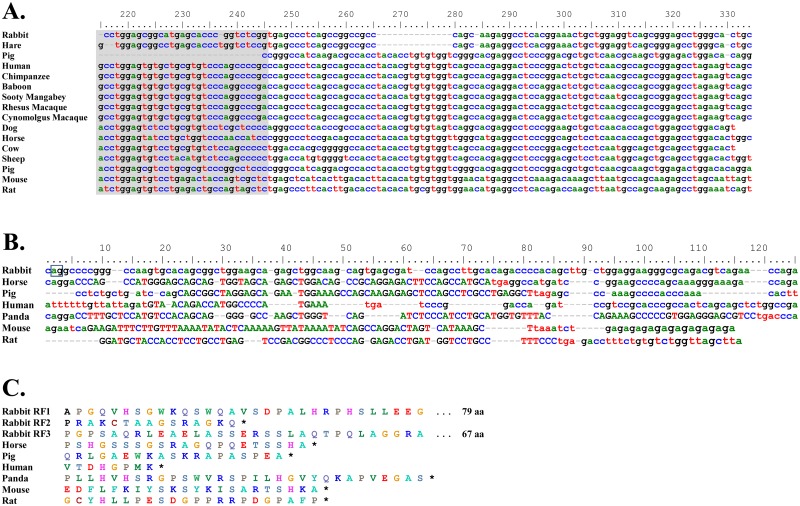
Rabbit remnant *δCH3* and *Cδ* secretory (*δS*) exons. A) Alignment of rabbit and hare remnant *δCH3* exons with 3’ end of *δCH3* exons of various mammalian species. The region with no similarity to other mammalian *δCH3* exons is highlighted in gray. B) Alignment of rabbit remnant *δS* exon with *δS* exons of various mammalian species. Translated region indicated by uppercase letters. Potential 5’ splice site in rabbit *δS* (shared with horse and panda) enclosed in box. C) Amino acid sequence alignment of rabbit and other mammalian *δS* exons. RF1, RF2 and RF3 indicate the three potential reading frames for rabbit *δS* (only the first 31 amino acids of RF1 and RF3 shown and total length is indicated). Asterisks indicate stop codons. Nucleotide positions in A) and B) are numbered above each panel; dashes indicate nucleotide gaps.

**Fig 4:**
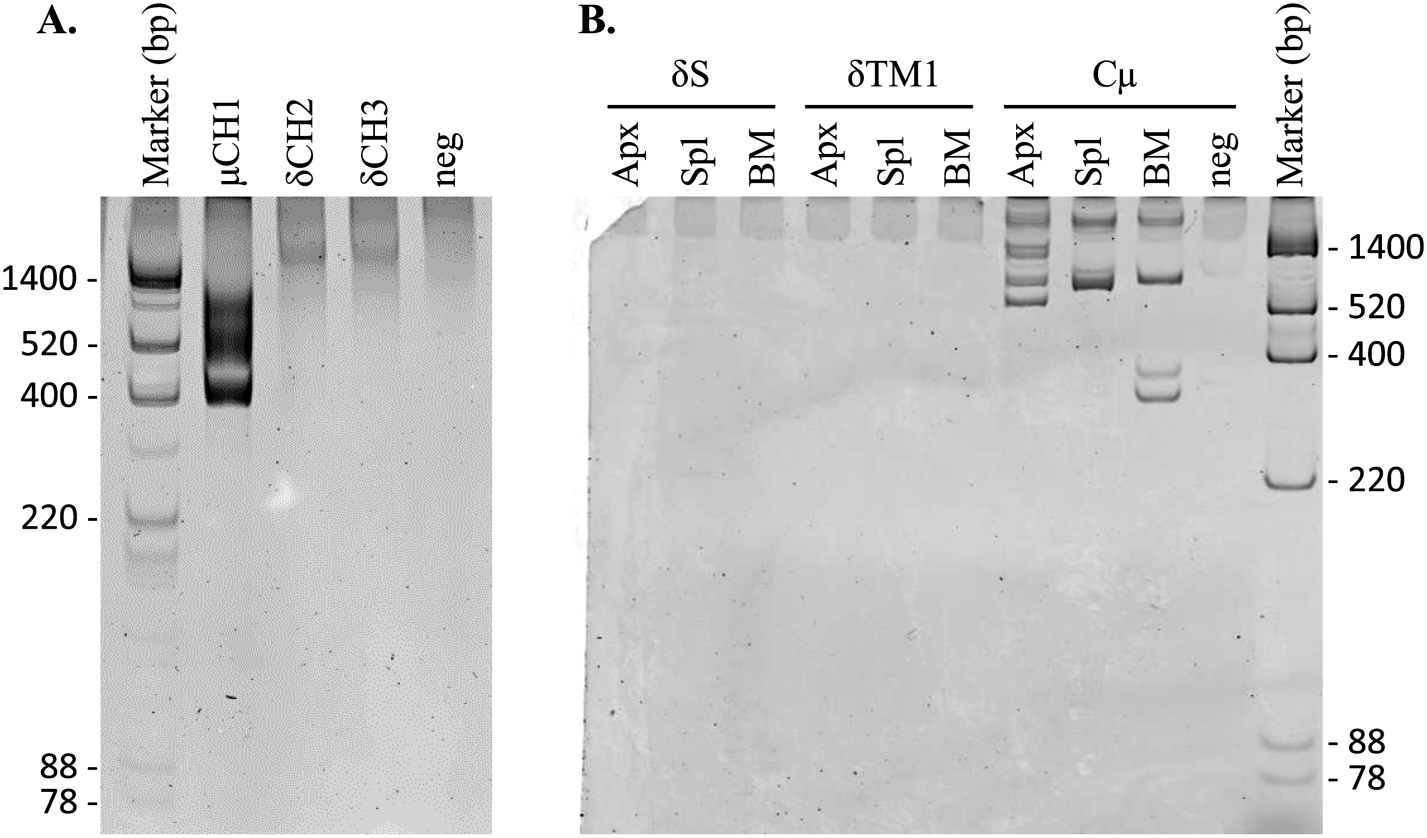
RT-PCR and 3’RACE analysis of the rabbit remnant *δCH2*, *δCH3*, *Cδ* secretory (*δS*) and *Cδ* transmembrane 1 (*δTM1*) exons. A) RT-PCR analysis of rabbit *δCH2* and *δCH3*. RT-PCR was performed using a forward primer in *V*_*H*_*1* (the preferentially rearranged rabbit *V*_*H*_ gene segment) and reverse primers in *Cμ* (lane 2; positive control), *δCH2* (lane 3), or *δCH3* (lane 4). Lane 5 (neg), negative control (same primers as lane 2, with no template). cDNA prepared from appendix of a 6-week-old rabbit. Forward primers: VDJ-Cμ Nest f2; reverse primers: VDJ-Cμ Nest r2, RbDelta CH2 r2, RbDelta CH3 r; Marker band sizes indicated in base pairs (bp). B) 3’RACE analysis of *δS* and *δTM1*. 3’RACE was performed for *δS* (lanes 1–3), *δTM1* (lanes 4–6) and *Cμ* (lanes 7–9; positive control) using cDNA prepared from appendix (Apx), spleen (Spl) and bone marrow (BM) of a 6-week-old rabbit. Forward primers: RbDelta SecExon 3’RACE f1, RbDelta SecExon 3’RACE f2, RbDelta TM1 3’RACE f1, RbDelta TM1 3’RACE f2, Cmu f, Mid mu f; Lane 10 (neg), negative control (same primer pair as lanes 7–9, with no template). Marker band sizes indicated in base pairs (bp). 3’RACE primers and protocol as described in [33].

Although much of the ancestral *Cδ* gene is still present, the exons have been mutated and pseudogenized. Comparison of the rabbit *δCH2* exon with that of other mammals, for example, revealed that, despite a clear overall similarity, rabbit *δCH2* has been disrupted by nucleotide insertions and deletions (Fig 2). Most strikingly, the entire 3’ one-third of the exon is missing. The rabbit *δCH3* exon is much more extensively disrupted, with only about the 3’ one-quarter of the exon remaining (Fig 3A). It is not clear how the 3’ and 5’ regions of *δCH2* and *δCH3*, respectively, were lost. The intervening region between them contains a 369 nt truncated LINE (L1MA9 5) that lies 57 nt downstream of the *δCH2* remnant and overlaps ~70 nt of the expected 3’ end of a full-length *δCH2* exon (Figs 1 and 2). It is not a typical retrotransposon insertion, which would be flanked by short direct repeats and formerly contiguous genomic DNA sequence. LINE insertions, however, are also associated with deletion of target DNA sequence [44–46]. The region between the LINE and the *δCH3* remnant contains many duplicated sequences with homology to the LINE, the largest ranging in size from 125–233 nt in length, with far more numerous shorter repeated sequences (S2 Fig). Because LINEs can undergo internal rearrangements during genomic insertion associated with large deletions [44], it is possible that the 3’ end of *δCH2* and the 5’ end of *δCH3* were lost during LINE insertion-mediated deletion of target DNA sequence. The loss of functional *δCH2* and *δCH3* exons would have rendered the entire *Cδ* gene nonfunctional. Supporting the conclusion that the rabbit *δCH2* and *δCH3* exons are non-functional remnants, we found no evidence that either are utilized in VDJ gene transcripts by RT-PCR using forward primers in *V*_*H*_*1* (the preferentially rearranged rabbit *V*_*H*_ gene segment) (Fig 4A).

To determine when, during lagomorph evolution, the *Cδ* gene became non-functional, we asked whether the *δCH2* and *δCH3* exons were similarly disrupted in other lagomorphs. The first major divergence during lagomorph evolution was the divergence of the pika (family Ochotonidae) from the lineage that led to the rabbits and hares (family Leporidae) ~35–55 million years ago [47]. The subsequent divergence of rabbits and hares occurred ~12 million years ago [48–50]. We therefore attempted to PCR-amplify the genomic region containing the *δCH2* and *δCH3* exons, and the intervening region between them, from Iberian hare (*Lepus granatensis*) and American pika (*Ochotona princeps*) genomic DNA. We found that *δCH2* and *δCH3* were also disrupted in *L*. *granatensis* genomic DNA (Figs 2 and 3A), but we were unable to PCR-amplify this region from *O*. *princeps* genomic DNA. These results strongly suggest that the *Cδ* gene became non-functional in lagomorphs before the divergence of rabbits and hares ~12 million years ago.

**Fig 5:**
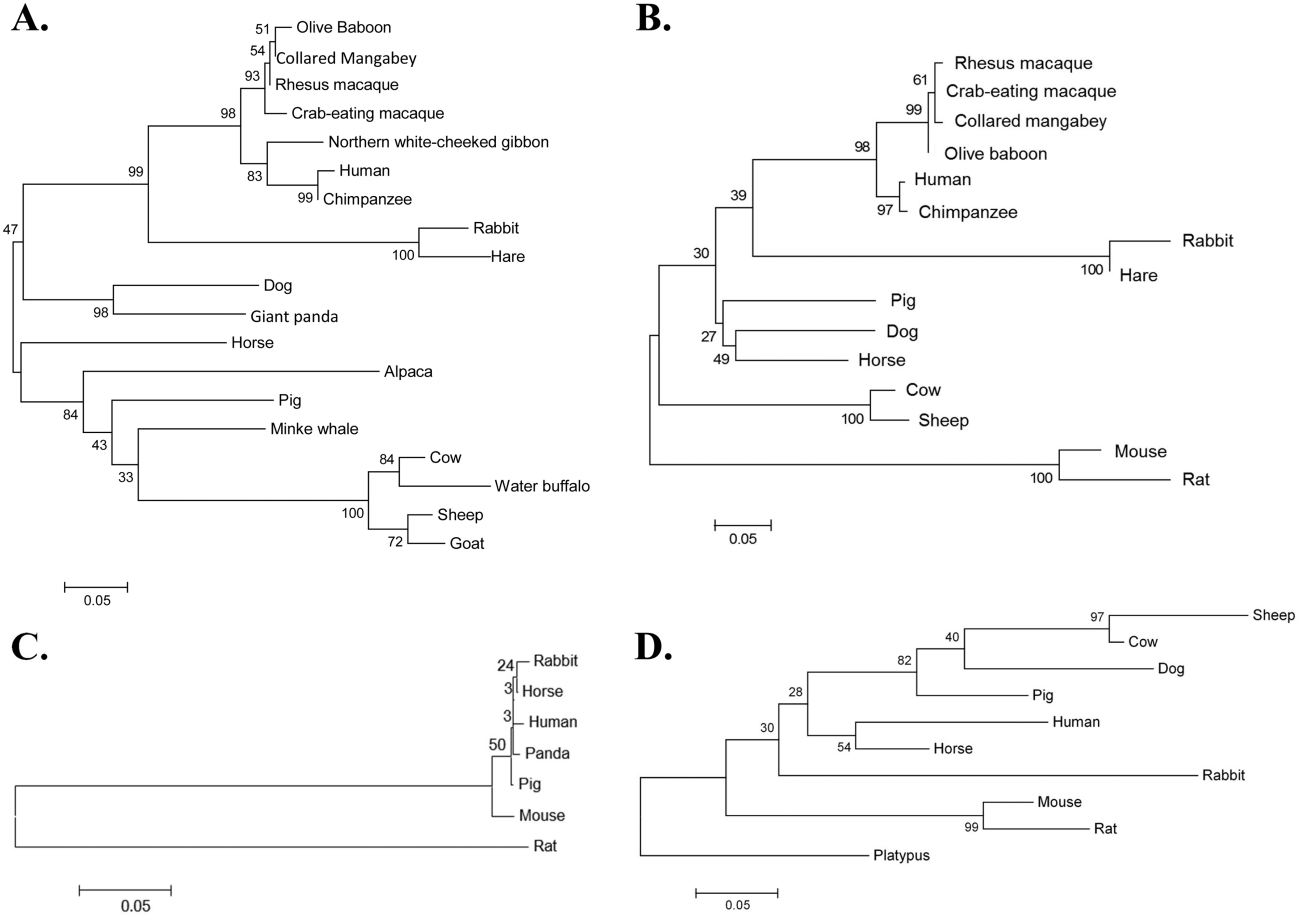
Maximum likelihood phylogenetic trees for mammalian *Cδ* exons. A) *δCH2*, B) *δCH3*, C) *Cδ* secretory exon, D) *Cδ* transmembrane exon 1. Platypus included as an outgroup in D. Bootstrap values from 1000 replicates appear next to the nodes.

We estimated the extent of genetic divergence between the lagomorph *δCH2* remnant and the *δCH2* exons of other mammals by calculating the genetic distances between them using the General Time Reversible model. These results are presented diagrammatically as a maximum likelihood phylogenetic tree in Fig 5A. The lagomorph *δCH2* remnants were most closely related to the *δCH2* exons of human and other primates. The lagomorph and primate cluster was in turn most closely related to the *δCH2* exons of the dog (*Canis familiaris*) and giant panda (*Ailuropoda melanoleuca*). In these analyses, three matches with uncharacterized mRNA from the Minke whale (*Balaenoptera acutorostrata*, *ssp*. *scammoni*), the northern white-cheeked gibbon (*Nomascus leucogenys*) and the water buffalo (*Bubalus bubalis*) likely indicate that these are IgD mRNA from these species. Similar analyses of the leporid *δCH3* remnants suggested they are also most closely related to the *δCH3* exons of human and other primates, and much more distantly related to those of mouse and rat (rodents were not included in the *δCH2* analyses because they lack *δCH2*) (Fig 5B).

### The rabbit Cδ secretory (δS) exon

The rabbit *δS* remnant was identified in a BLAST search as a 73 nt region with 72% identity to the horse (*Equus caballus*) *δS* (Fig 3B). The rabbit *δS* remnant was located 3934 nt downstream of the 3’ end of the *δCH3* exon remnant, comparable to the location of the *δS* exon in the *Cδ* loci of other vertebrates (Fig 1). Phylogenetically, the rabbit *δS* remnant clusters tightly with the *δS* of horse, pig, human and panda (Fig 5C). These in turn are less closely related to the mouse *δS* and far more distantly related to that of the rat.

Despite the short genetic distances between horse, pig, human and panda *δS* at the nucleotide level, they encode considerably different amino acid sequences due to the use of different splice sites and reading frames, as well as nucleotide insertions and deletions (Fig 3B and 3C). Alignment of the rabbit *δS* nucleotide sequence with the *δS* of these mammals, as well as mouse and rat, revealed that the splice site utilized by horse and panda *δS* is present in the rabbit sequence (Fig 3B, boxed). While the first and third reading frames potentially utilizing this splice site encode extremely long amino acid sequences (79 and 67 amino acids in length, respectively), the second encodes a 15 amino acid sequence, comparable in length to those of other mammals (Fig 3C). We therefore considered the possibility that the rabbit *δS* might encode a functional secretory terminus, possibly capable of alternative splicing onto *Cμ* transcripts. We tested this possibility by 3’RACE, using primers within the rabbit *δS* nucleotide sequence and cDNA prepared from rabbit bone marrow, spleen and appendix, and found no evidence of transcripts utilizing the rabbit *δS* (Fig 4B)). These results strongly suggest that *δS* is a non-functional remnant exon.

### The rabbit Cδ transmembrane (δTM1 and δTM2) exons

**Fig 6:**
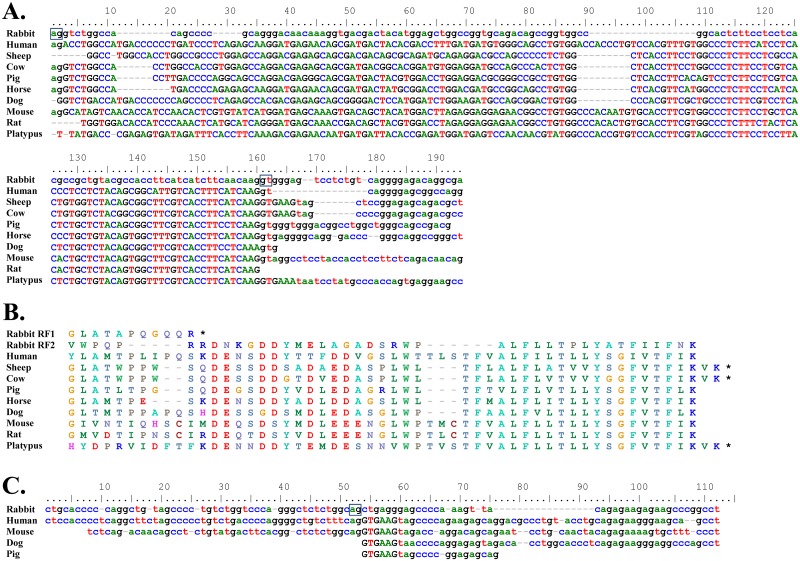
Rabbit remnant *Cδ* transmembrane (*δTM1* and *δTM2*) exons. A) Nucleotide sequence alignment of rabbit remnant *δTM1* exon with *δTM1* exons of various mammalian species. Potential 5’ and 3’ splice sites in rabbit *δTM1* enclosed in boxes. B) Amino acid sequence alignment of rabbit and other mammalian *δTM1* exons. RF1 and RF2 indicate reading frames 1 and 2 for rabbit *δTM1*. Asterisks indicate stop codons. C) Nucleotide sequence alignment of rabbit remnant *δTM2* exon with *δTM2* exons of various mammalian species. Potential 5’ splice site in rabbit *δTM2* enclosed in box. Translated region in A) and C) indicated by uppercase letters. dashes, nucleotide or amino acid gaps.

The first rabbit *Cδ* transmembrane exon (*δTM1*) lies 1580 nt downstream of the *Cδ* secretory exon remnant (Fig 1). It was identified in a BLAST search as a 139 nt region with 68% identity to the horse *δTM1* (Fig 6A). As shown in the phylogenetic tree in Fig 5D, it clusters most closely with the *δTM1* of horse and human, and somewhat less closely with the *δTM1* of pig, dog, cow and sheep. Among the mammals examined, the rabbit *δTM1* is least closely related to those of mouse and rat.

Alignment of the rabbit *δTM1* nucleotide sequence with those of other mammals showed that the rabbit sequence contains nucleotide gaps probably resulting from genetic decay, rather than codon insertions or deletions as seen in other mammals (Fig 6A). Although potential 5’ and 3’ splice sites are present in rabbit *δTM1* (Fig 6A, boxed), the presumed reading frame (RF1) contains a frameshift resulting in a premature stop codon (Fig 6B). Although reading frame 2 (RF2) yielded an amino acid sequence broadly similar to *δTM1* of other mammals (Fig 6B), rabbit *δTM1* appears nonetheless unlikely to encode part of a functional transmembrane terminus because the second transmembrane exon (*δTM2*) is clearly non-functional.

The *δTM2* of all mammals examined encodes only two amino acids, valine (V) and lysine (K). In sheep, cattle and platypus, valine and lysine codons occur at the end of *δTM1* and are followed by a stop codon (Fig 6A), suggesting that these species might encode the entire *Cδ* transmembrane terminus with *δTM1*. The rabbit *Cδ* locus appears, as in most mammals, to have encoded the two terminal amino acids of the transmembrane region on a second *δTM* exon. The rabbit *δTM2* exon lies 187 nt downstream of *δTM1* (Fig 1). Although it contains a potential 5’ splice site (Fig 6C, boxed), the *δTM1* exon encodes leucine (CTG) and arginine (AGG) residues, rather than valine (GTG) and lysine (AAG or AAA) residues as observed in all other mammals and the two codons are not followed by a stop codon as seen in all other mammals (Fig 6C). The rabbit *δTM1* and *δTM2* exons therefore appear to be non-functional. Consistent with this conclusion, we found no evidence of their use in mRNA transcripts by 3’RACE in rabbit bone marrow, spleen or appendix cDNA (Fig 4).

## Discussion

The absence of a rabbit IgD gene immediately downstream of the IgM gene has previously been reported [7, 8]. Here, we demonstrated that much of the rabbit IgD gene is still present in the Ig heavy chain locus as a pseudogenized remnant located ~21 kb downstream of the IgM gene. The remnant locus has lost the *δCH1* and hinge exons, but the secretory and transmembrane exons, as well as portions of the *δCH2* and *δCH3* exons, have been retained. We found no evidence, by RT-PCR and 5’ and 3’ RACE, that any of the remnant exons are transcribed, and we therefore conclude that the rabbit *Cδ* locus is completely non-functional.

Although it is not possible to determine how the rabbit *Cδ* locus became non-functional, the remnant locus suggests three potential explanations: the loss of the *δCH1* and hinge exons, disruption of the *δCH2* and *δCH3* exons and displacement of the locus from proximity to the *Cμ* locus by the insertion of multiple repetitive DNA elements. There is evidence from other species that the *Cδ* locus can survive the loss of *δCH1*. Indeed, the *Cδ* locus of teleost fish does not contain a *δCH1* exon and the *μCH1* exon is instead utilized in *Cδ* transcripts [18]. The *δCH1* sequence in *Cδ* cDNA from the pig is identical to *μCH1*, suggesting that the pig might use a similar strategy to produce IgD [23, 51]. While the δCH1 domains of cattle and sheep IgD are nearly identical to their respective μCH1 domains, the δCH1 domain is encoded by a *δCH1* exon in cattle and probably also in sheep [23]. In both species, the *δCH1* exon and its 5’-flanking sequence appear to have been duplicated from a region of the IgH locus spanning the intronic enhancer and the *μCH1* exon. Zhao *et al*. (2002) reported evidence that this duplication event occurred in the common ancestor of cattle and sheep, either replacing an existing *δCH1* exon or restoring a *δCH1* exon that had been deleted or damaged [23]. Their results further suggested that a gene conversion event probably occurred between the *μCH1* and *δCH1* exons in both cattle and sheep following speciation. Taken together, these results demonstrate that the *Cδ* locus can compensate for the loss of *δCH1*. Nonetheless, the manner in which rabbit *δCH1* was lost, possibly with the *Cδ* hinge exons, might have precluded alternative splicing of the remaining *Cδ* locus to *μCH1*. In the absence of a fortuitous duplication event that could restore *δCH1*, as appears to have occurred in cattle and sheep, the *Cδ* locus would have become non-functional.

The disruption of the *δCH2* and *δCH3* exons could also have disabled the rabbit *Cδ* locus. Although mice and rats produce functional IgD despite lacking a *δCH2* exon [52, 53], the loss of 30% of the 3’ end of *δCH2* and 74% of the 5’ end of *δCH3*, possibly through LINE insertion-mediated deletion, would preclude the generation of a functional *Cδ* transcript. Another possibility is that multiple repetitive DNA element insertions displaced the *Cδ* locus far enough downstream of the *Cμ* locus to prevent its co-transcription, causing it to fall into disuse and decay. At least 19 DNA element insertions, collectively comprising about 16 kb of DNA, occurred in the region between *μTM2* and the *δCH2* remnant. Consequently, this region is twice as large (21 kb) as the corresponding region of the human heavy chain locus. In addition to disrupting genetic loci through insertion, retrotransposons can delete host DNA sequences, either during integration or by providing regions of sequence homology, after integrating, that promote intra- and inter-chromosomal recombination events [44, 46, 54]. Given the high density of repetitive elements between *μTM2* and the *δCH2* remnant, *δCH1* and the hinge exons might have been lost through such a mechanism.

Lagomorph evolution is marked by two major divergences, that of the pika lineage from the rabbit/hare lineage ~35–40 mya [47], and the divergence of rabbits and hares ~12 mya [48–50]. We found that *δCH2* and *δCH3* are disrupted in the Iberian hare nearly identically to *δCH2* and *δCH3* of the European rabbit. This result strongly suggests that the lagomorph *Cδ* locus became non-functional prior to the divergence of rabbits and hares. We cannot rule out the possibility that hares possess functional *δS* and/or *δTM* exons that are alternatively spliced into *Cμ* transcripts or utilized in unusual IgD transcripts comprised solely of a δCH1 domain, but the likelihood of these scenarios is remote. We were not able to determine whether *δCH2* and *δCH3* are similarly disrupted in the pika, despite using a sensitive touchdown PCR approach. Our results therefore suggest that the loss of lagomorph IgD occurred sometime prior to the divergence of rabbits and hares ~ 12 mya.

The loss of IgD, however, occurred in lagomorphs after their divergence from rodents, because both the mouse and rat possess functional *Cδ* loci [52, 53]. Lagomorpha and Rodentia form the monophyletic group Glires, and are estimated to have diverged between 61.5 and 100.5 mya [55, 56]. Glires is a sister group of Euarchonta (treeshrews, colugos and primates), with which it comprises the superorder Euarchontoglires [57–59]. Following their divergence from lagomorphs, rodents underwent an extraordinary adaptive radiation, accompanied by accelerated evolutionary rates, such that they currently represent nearly half of all mammalian species. Consistent with the rapid evolutionary divergence of rodents within the Euarchontoglires, the rabbit remnant *Cδ* exons are most closely related to the corresponding *Cδ* exons of primates, and most evolutionarily distant from those of the mouse and rat. Similarly, several additional immunologic genes have been reported to exhibit closer evolutionary relatedness between human and rabbit than between either and mouse [60, 61]. Indeed, the evolutionary distance of the rabbit remnant *Cδ* exons from mouse and rat *Cδ* exons consistently exceeded that from species such as the horse, dog, sheep and pig, which belong to the superorder Laurasiatheria, a sister group to Euarchontoglires.

It is not clear why previous studies found evidence suggesting that IgD is expressed by rabbit B cells. These studies relied on the removal of IgM, either through immunoprecipitation of cell lysates or capping of B cells with anti-IgM Abs, and subsequent identification of residual immunoglobulin molecules that did not react with anti-IgM, -IgG or -IgA Abs [15–17]. Because it is now clear that the residual immunoglobulin molecule identified in these studies was not IgD, one or more of the isotype-specific Abs used in these studies apparently did not detect all the molecules within its isotype specificity. Further studies would be necessary to determine the reason for this.

IgD is one of the evolutionarily oldest IgH isotypes, first appearing (as its ortholog, IgW), along with IgM, in the cartilaginous fishes [3]. It has persisted throughout subsequent vertebrate evolution and is present in all fish, amphibians, nonavian reptiles, and most mammals, examined. Although birds were thought to lack IgD due to its absence in chickens and ducks [4, 5], Han *et al*. (2016) recently identified a functional IgD gene in ostriches and, by surveying bird genomes, found *Cδ* genes in 18 bird species from 12 orders [28]. While its early evolutionary origin and sustained presence in all orders of jawed vertebrates suggest that IgD serves an important immunologic role, its absence in some mammalian and bird species appears inconsistent with this view. Perhaps, as suggested by Ohta and Flajnik (2006)[3], IgD has been retained during vertebrate evolution as a versatile complementary isotype to IgM, serving varying functions in different vertebrate taxa. These authors note that, unlike IgM, IgD exhibits considerable variability, both in structure and expression, among vertebrates. The number of IgD constant domains, for example, ranges from 2 in mice to 19 in the Siberian sturgeon [52, 62] and IgD is differentially expressed as a primarily secretory or transmembrane molecule, or in multiple alternatively spliced forms, among vertebrates. In the African coelacanth, which lacks an IgM-encoding gene, IgD (IgW) gene might have provided redundancy against the loss of IgM, which is critical for early B cell development and forms the primary antibody repertoire [63]. Although the role of IgD in B cell function remains unclear, some unique roles have recently been described in mice and humans. In mice, for example, IgD minimizes tolerance-induced holes in the pre-immune Ab repertoire by attenuating anergic B cell responses to self-antigen [64]. Further, due to inherent structural differences between the IgM and IgD hinge regions, the two isotypes differ in their ability to activate B cells [66]. While IgM B cell receptors are responsive to both mono- and polyvalent antigens, IgD B cell receptors respond only to polyvalent antigens [65]. Thus, the relative expression levels of IgD and IgM skew B cell responses to polyvalent antigens, such as immune complexes, or monovalent antigens. In humans, circulating IgD interacts with basophils through a calcium-fluxing receptor that induces antimicrobial, opsonizing, inflammatory and B cell-stimulatory factors [66]. Whether IgD plays similar, or additional, roles in other species remains to be determined. It is nevertheless clear that some vertebrate species can tolerate the loss of IgD. Our findings demonstrate that the rabbit IgD locus is pseudogenized and non-functional, and that lagomorphs probably lost the ability to express IgD at some point prior to the divergence of rabbits and hares ~ 12 mya.

## Supporting information

S1 Fig Putative σδ switch region in the rabbit Cμ-Cδ intron. TGGGG, TGGGC and TGAGC pentameric repeats are capitalized. The nucleotide composition of the region is A: 177, C: 277, G: 357, T: 178. The region’s location in the Cμ-Cδ intron is indicated in Fig 1.
(TIF)

S2 Fig Harr plot of intervening genomic sequence between *δCH2* and *δCH3* remnant exons. Distances, in nucleotides, are indicated on the x and y axes. The positions of *δCH2*, *δCH3* and L1MA9_5 are indicated below the plot.
(TIF)
