# Supplementary figures and images for: The remnant of the European rabbit (Oryctolagus cuniculus) IgD gene
Source: PLoS One. 2017 Aug 23;12(8):e0182029. doi: 10.1371/journal.pone.0182029 (PMC5568218; doi:10.1371/journal.pone.0182029)

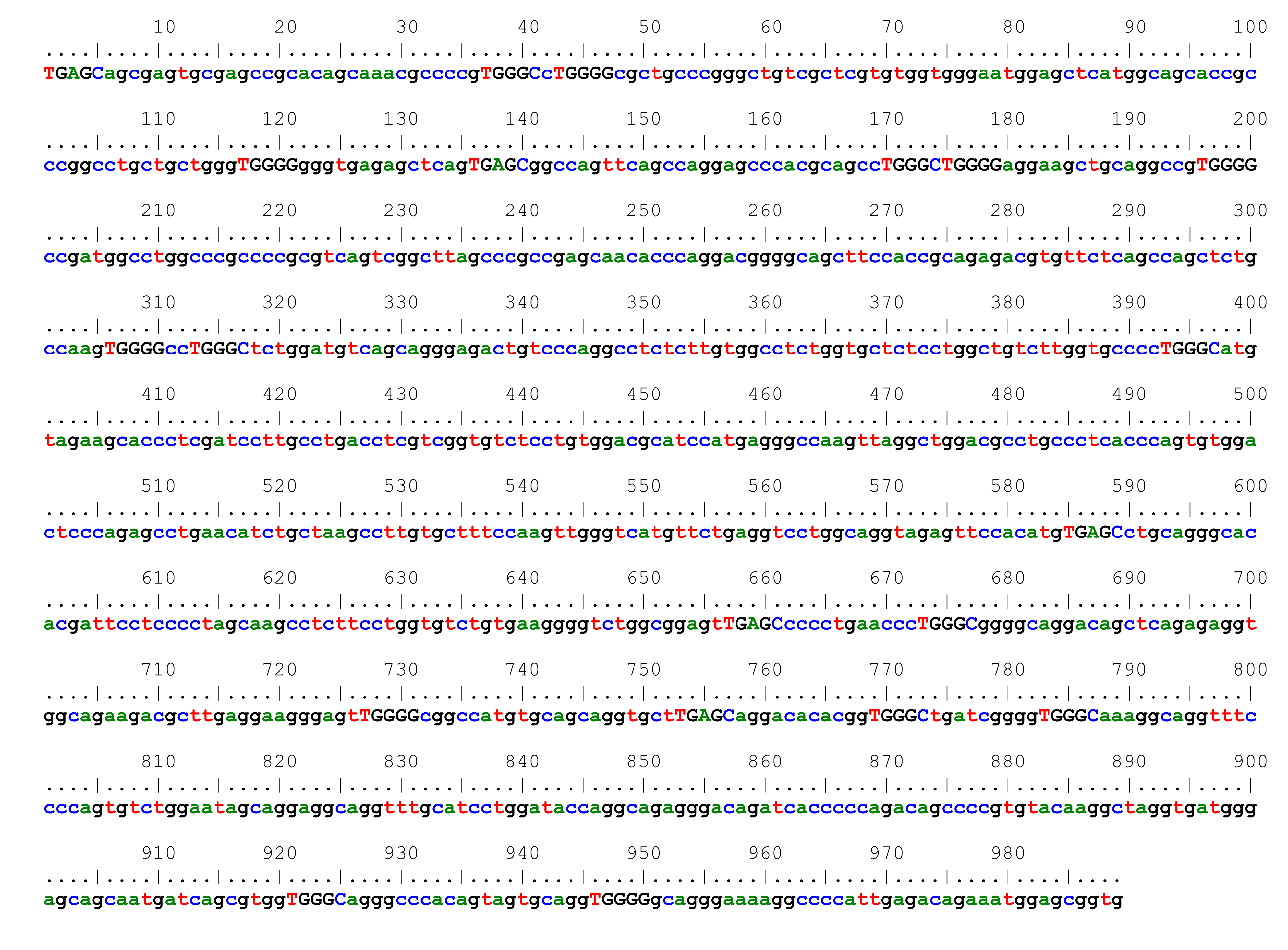

Supplement: S1 Fig — TGGGG, TGGGC and TGAGC pentameric repeats are capitalized. The nucleotide composition of the region is A: 177, C: 277, G: 357, T: 178. The region’s location in the Cμ-Cδ intron is indicated in Fig 1. (TIF) [file pone.0182029.s001.tif]

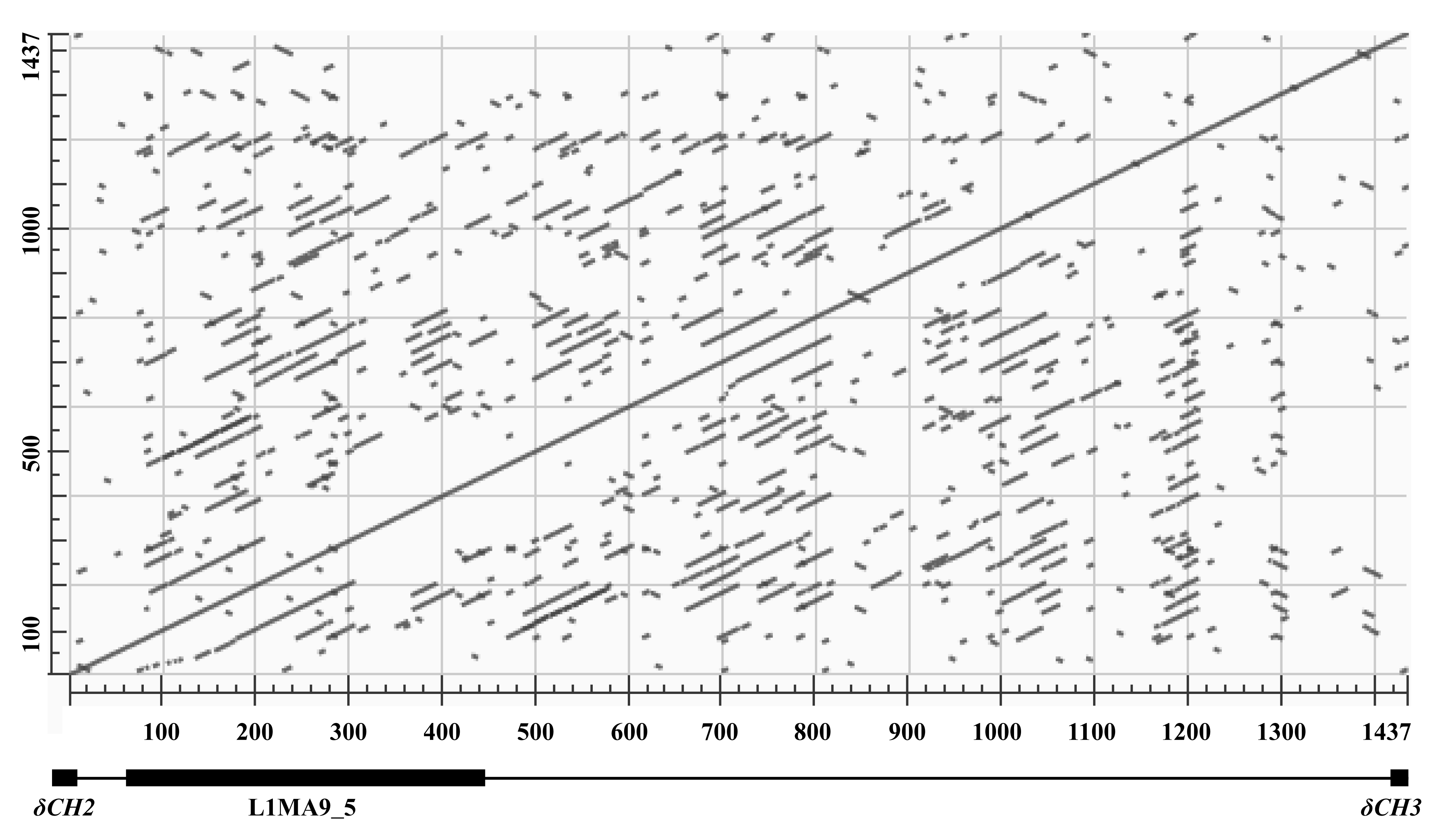

Supplement: S2 Fig — Distances, in nucleotides, are indicated on the x and y axes. The positions of δCH2, δCH3 and L1MA9_5 are indicated below the plot. (TIF) [file pone.0182029.s002.tif]
